# Fitting work? Students speak about campus employment

**DOI:** 10.1007/s12186-023-09333-y

**Published:** 2023-08-26

**Authors:** Alison Taylor, Catalina Bobadilla Sandoval

**Affiliations:** 1 Department of Educational Studies, University of British Columbia, Education Centre at Ponderosa Commons, 6445 University Boulevard Vancouver, British Columbia, V6T 1Z2 Canada; 2 Department of Educational Studies, University of British Columbia, Vancouver, Canada

**Keywords:** Campus employment, Workplace experiences, Learning cultures

## Abstract

Balancing part-time work and studies has become commonplace for university students in Canada and other countries where the costs of education have risen over time. While there is a substantial literature on the impacts of term-time work on studies, little has been written about campus employment programs, which are becoming more commonplace in North American universities. This paper addresses this gap by considering students’ experiences in such a program at a western Canadian university. Focusing primarily on qualitative data from a longitudinal study, we examine the various reasons for the attractiveness of this program, which go beyond the promise of professional, career-related work experience. Our analysis draws on the academic literature on work-study roles, which examines whether term-time work has a more positive or negative effect on student outcomes as well as sociocultural literature that is more attentive to different contextual features of the work-study relationship. We find that university-sponsored jobs are highly valued by students for their workplace relationships, regulation, and flexibility. Positive relationships at work are facilitated by supervisors’ recognition of students’ academic priorities and opportunities to develop peer-support networks on campus. Other important features for students include the convenience of working where one studies, and the ability to build work schedules around academic schedules. However, the limited access to ‘good’ campus jobs raises concerns about equity.

## Introduction

The competition for professional jobs has increased with the massification of higher education globally (Brown et al., 2020). To facilitate graduates’ transitions to work, Canadian universities have expanded their work-integrated learning programs, including internship, practicum, cooperative education, field placement, and service learning (Jackson, 2015). Campus employment programs also aim to facilitate students’ transitions. Beyond providing financial support for students, these programs promise to offer high quality, enriching part-time jobs that provide meaningful learning and facilitate students’ transitions to graduate employment. Further, they tend to be highly regulated and usually provide more flexibility than off-campus jobs.

Based on our longitudinal mixed-methodology study at a large western Canadian university, this paper examines the question of how different students experience such programs. In particular, we consider whether campus employment is seen by students as more attractive than work off-campus, and how they think about this work in relation to their studies. Our analysis is informed by the academic literature on work-study roles, which examines the effects of term-time work on academic outcomes (e.g., Butler, 2007), as well as sociocultural learning literature, which attends more to contextual features of the work-study relationship (Hodkinson et al., 2007). Survey data from our study suggest that although campus jobs make up a small proportion of student jobs, participants describe their work conditions more favorably than other working students. Longitudinal, qualitative data from thirty undergraduates provide a more nuanced perspective, which highlights the differences as well as commonalities in student jobs and experiences. This paper provides insights into how students perceive these positions, including congruence with their career interests, workplace relationships, and the importance of regulation and flexibility.

This research contributes to the slim academic literature on campus employment programs and informs broader discussions about the importance of regulation and flexibility in student work. We find that campus positions are attractive to students beyond their promises of building professional skills. Such jobs are attractive primarily because of their worker-centred flexibility and regulation, and are especially valuable when students experience supportive relationships with co-workers and employers. We conclude with suggestions for how campus employers and universities can further realize the potential of such programs.

## Literature review and conceptual frame

### On-campus employment programs

In campus employment programs, which are co-curricular, student workers are required to take responsibility for making connections between learning in classrooms and on the job. They are usually expected to direct and manage their learning with varying levels of support from supervisors and program staff. Thus, students tend to be positioned toward the ‘worker’ end of the work-study continuum. At the same time, since positions are part-time, short-term, and undertaken alongside full-time studies, employers are expected to accommodate students’ timetables and support them in prioritizing their studies. In contrast, the integration of classroom and workplace learning is usually built into work-integrated learning (WIL) programs like practicum (Billett, 2015).

The literature on WIL programs like cooperative education (cf. Kramer & Usher, 2011 in Canada) is much larger than research on campus employment programs coordinated by universities. One of the few studies on the latter is sponsored by the National Association of Student Personnel Administrators (NASPA) on programs in the U.S. (Burnside et al., 2019). This report suggests that on-campus jobs in public four-year institutions are most commonly found in residence life, followed by recreation services and fitness centers, and academic schools and departments. The most common program goal is to improve students’ financial security. Writing for a practitioner audience, authors recommend that programs prioritize student learning by identifying the competencies and employability skills to be gained from campus jobs, provide students with opportunities to reflect on their learning, and hold regular feedback sessions with employers (Burnside et al., 2019). The importance of ensuring equal opportunities for students to apply to positions is also emphasized.

Similarly, research on a campus employment program at a large Canadian university argues that more structured learning support is needed to enhance students’ professional development and workplace performance (West & Stirling, 2021). Based on a survey of 716 student participants and a smaller sample of employers, authors make connections between structured learning support and positive student outcomes. In particular, student employees who completed goal setting and reflection exercises with their workplace supervisor are more likely to make connections between their job and academic studies, to see their work as meaningful, and to see it as increasing their awareness of their skills and/or strengths. Authors recommend that universities do more to provide high quality professional development opportunities for students. These findings and recommendations are echoed by Billett (2015) who suggests that workplace experiences need to be augmented by helping students clarify the applicability of what they learn in practice-based experiences.

### Flexibility, security, and regulation in work

Although our focus in this paper is on-campus employment, most Canadian post-secondary students work in the low-wage service sector (Marshall, 2010). In 2018, the highest proportion of minimum wage workers in Canada were employed in retail trade (33%) and accommodation and food services (26%) (Dionne-Simard & Miller, 2019). In these jobs, schedules are commonly unpredictable, and workers lack control over tasks and work pace. While low-wage workers are more reliant on regulation of wages and conditions (Carré & Tilly, 2012), employers’ noncompliance with standards is also greater in this sector (Vosko et al., 2016). Areas of labour standard violations typically include failure to pay overtime, lack of paid sick days, and failure to provide rest and meal breaks (Bernhardt et al., 2008). The workers most affected by the erosion of labour standards are typically those who have “little or no recourse to either challenge an employer’s behaviour or to seek employment elsewhere” (Bernhardt et al., 2008, p. 21). University students, who often see themselves as temporary members of the low-wage job market and lack experience, are more vulnerable and susceptible to exploitation at work (cf. Tannock & Flocks, 2003). Further, the concentration of students in some areas of low-wage work may have the unfortunate effect of reducing the pressure on employers to improve wages and working conditions (Lloyd & Payne, 2016).

In comparison, campus jobs might be expected to be more regulated and flexible, features seen as beneficial for employees’ sense of autonomy and well-being. Kossek and Lautsch (2018) define flexibility as “employment-scheduling practices that are designed to give employees greater work-life control over when, where, for how long, or how continuously work is done” (p. 10). Hill et al. (2008) consider to what extent flexible work arrangements are motivated by organizational needs or employee needs. In the latter case, the work is “designed to prioritize the ability of workers to self-regulate work-related responsibilities” (p. 151); flexibility is worker-centred (Chung et al. 2013).

These findings are relevant for research into students’ term-time work, a topic that has been largely ignored in research on flexibility and work. Worker-centred flexibility is important for students who tend to be time pressured; in response to growing expectations and demands, they tend to “jealously guard and manage their time, including that allocated to their studies” (Billett, 2015, p. 147). Ideally then, term-time work fits neatly around students’ academic schedules, offers the desired number of hours per week, affords the ability to exit and enter the workforce (if needed), and allows students to manage unexpected personal responsibilities (cf. Hill et al., 2008). This ideal is more likely to be realized in campus employment programs, where employers are expected to accommodate student timetables.

Regulation in such programs includes screening of job postings by program staff, adherence to university pay scales with annual increases, and the requirement that applications for positions address student learning. Participants may thus have access to forms of flexibility and regulation (including remote work during the pandemic) that are associated with higher level occupations. On the other hand, if students are working with professional employees who are facing intensification in their work (Kelliher & Anderson, 2010), they may be adversely affected too. Given the paucity of research, it is important to empirically explore whether campus employment programs help or hinder participants’ ability to prioritize their studies (Kossek & Lautsch, 2018).

### Social relations at work and school

Two other strands of academic research are important for our analysis: the first examines work-study roles, and the second emphasizes the importance of work and learning contexts. An example of the first strand is the model of work-study conflict and work-study facilitation developed by Butler (2007) and adopted in other quantitative research studies (e.g., Park & Strung, 2013). Work-study facilitation (WSF) occurs when the demands of the student role are compatible with those of the work role. For example, work factors like role clarity, skill variety, and autonomy are found to be positively correlated with study time (Barling et al., 1995, as cited in Butler, 2007). In contrast, work-study conflict (WSC) is evident when work requires time away from school-related activities or creates strain that impedes school performance. Support from supervisors and co-workers can enhance WSF and reduce WSC (Wyland et al., 2016). Other researchers argue that aspects of conflict and facilitation coexist in work and study relationships, and the antecedents of work-study relations must be considered (Cinamon, 2016). Such antecedents include financial pressures on students, which are likely to affect the extent to which they have the necessary time to fully engage with the work placement and studies (Billett et al., 2018). Since campus employment programs aim to help students develop professional skills through meaningful employment, we might expect work-study roles to be more congruent for students in these jobs. They may therefore exemplify what is required for WSF and contribute to our understanding of that concept.

The second strand of research is critical for our qualitative data analysis because it attends to the importance of context in research on work-study relationships. Hodkinson et al. (2007) posit that understanding learning at work requires attention to various dimensions of what they call “learning cultures.” These dimensions include the positions, dispositions, and actions of students and others in the worksite; the location and resources of the site; policies and regulations in the site; the wider academic cultures of which the site is part; and wider social and cultural values and practices. We share the authors’ premises about learners and learning (Hodkinson et al., 2008) as follows:

- Learners are shaped by and shape practices in their worksite;
- Learners are embodied social beings who are socially positioned;
- Learning is influenced by wider social structures; and
- Learning involves relations of power.

Data from our study allow us to examine how students describe the relationship between their term-time work and studies, and more generally, their university experiences over time. It is clear that the learning of individuals is a process of “continual becoming, through participation in several different learning cultures over time” (Hodkinson et al., 2007, p. 425). As well as providing insight into the range and types of campus jobs and student employees, our analysis considers the question of how to enhance the likelihood of valuable learning within campus employment programs, given this diversity. We pay particular attention to how students’ workplace relationships and work arrangements affect their academic experience and vice-versa.

## Methodology and context

The Hard Working Student research project is a longitudinal, mixed-methodology study. The first phase of our research involved surveys of undergraduate students at a large western Canadian university in 2018 and 2019. 1,732 students completed the 2018 survey, and 2,987 students completed it in 2019. Respondents included working and non-working students. The surveys were followed by systematic longitudinal qualitative data collection conducted between 2019 and 2021 at the same university. 57 second year undergraduate students were recruited through the surveys, classroom visits, and university careers office listservs. Over three years, students were invited to participate in focus group (FG) interviews followed by life map (LM) sessions (where students recounted key work and study decisions since secondary school); audio diaries (AD) (where students participated in self-interviews to and from school and work); and follow-up interviews (FI). All 57 students in this study participated in at least two or more of these activities, and some participated in all of them. All data collection sessions were audio-recorded and fully transcribed. Ethical approval was obtained from the university ethics board for all phases of this research.

### Highlights from survey data

Our 2018 survey found that 9.7% of 2,987 undergraduate respondents participated in either campus employment placements or internships (the vast majority in the former) (Taylor et al., 2020). This is close to the findings from a 2014 national survey that only 11% of Canadian undergraduates worked on campus (CUSC, 2014). Women are over-represented among campus employment participants: 67% were involved although they made up 56% of undergraduates overall in 2018. International students were also over-represented: 34% were involved although they only made up 25% of undergraduates overall (Taylor et al., 2020). One explanation for the latter finding is that many international students seek job relevant work, particularly if they wish to immigrate to Canada after completing studies (Karim Jamal et al., 2023). While campus employment often involved jobs in research and development (38%), other students’ work occurred most often in retail or sales (24%), and accommodation or food (24%), according to survey data. Students participating in the campus employment program earned slightly higher wages than other working students.

In 2018, two-thirds of students involved in campus employment worked less than 11 h per week compared to around half of other students (Taylor et al., 2020). Non-campus employment is also reviewed by survey respondents less positively than these jobs. For example, 26% of students working in non-campus jobs strongly agreed that their job involved repetitive work compared to 18% of those involved in the campus employment program. Non-campus employment was also more likely to have variable schedules than campus jobs (62% vs. 50%). In our 2019 survey, more students in campus employment reported satisfaction with the atmosphere at work than other working students (37% vs. 23%). Further, 34% were satisfied with their job compared to 19% of others.

### The campus employment program

The campus employment program at this large western Canadian university aims to help students develop professional skills through meaningful employment. The program provides a wage subsidy to on-campus employers, who offer a diverse range of positions. Students work a maximum of 10 h per week during fall and winter semesters and up to 20 h per week in summer. Jobs are ideally, but not always, related to students’ fields of study and career aspirations. Institutional data suggest that common positions for undergraduates in campus employment for winter 2018 included project assistant (55.4% of postings), project worker (28.7%), office worker (8.3%), and researcher/professional (6.6%).

30 of our 57 participants in the qualitative phase of this study participated in campus employment program jobs. This paper focuses on the experiences of these participants, who include 17 domestic and 13 international students, 19 females and 11 males, 18 racialized and 12 white students. In comparison, our overall sample of 57 students included 41 domestic students, 39 females and 39 racialized students. Thus, it appears that international students were over-represented in campus jobs (43% campus jobs compared to 28% in our sample of 57), and there were fewer racialized students (60% campus vs. 68% in sample). Unlike our survey findings, there were also more young men (37% campus vs. 32% in sample). Pseudonyms are used for all participants. On average, these 30 students each partipated in three data collection activities. All 30 students were involved in focus groups, 25 participated in life map sessions, 13 completed audio diaries, and more than three-quarters participated in follow-up interviews.

## Thematic discussion

### The range of campus employment positions

Students who participate in the campus employment program in our study worked in a wide range of positions, from recreation centre staff to office assistant to technology and student support. The most common campus employment positions were in recreation and athletics (30%) and assistance and coordination (20%). It is noteworthy that all students in assistance and coordination positions were female, while two-thirds of students working in recreation and athletics were male. Institutional research is required to see if there is gender segregation in the program overall.

Half of the participants (15) speak of their work and school as connected, five are neutral, and 10 are explicit about the disconnect between their work and studies. The 15 students who perceive their jobs to be connected to their studies work in office, research, and project coordination. In comparison, 10 students working in recreation, athletics, and the library see their campus jobs as disconnected from studies, especially when they were majoring in unrelated areas. The group of five students who are neutral about whether their campus employment was connected to their studies or careers work in diverse on-campus jobs including tutoring, technology support, and recreation. They recognized that some work-tasks are helpful for their current studies but do not perceive an obvious connection. The work-study role literature suggests that when work and studies are seen as congruent, work is more likely to provide resources for students’ study role (i.e., the result is WSF) (Owen et al., 2017).

International students in this sample are evenly distributed between connected and disconnected jobs. More domestic students, female students, and racialized students report having jobs that are connected to their studies. However, our small sample size makes it difficult to generalize. In what follows, we attend to how students’ demographic characteristics and prior life experiences inform the way they make choices about work and how they access term-time jobs.

### Access to campus employment positions

Participants suggest that the campus employment program is very competitive, students often learn about it informally, students who gain positions feel lucky, and certain strategies seem to improve students’ chances of achieving positions. For example, the competitive nature of the program is evident from the experience of Ajay, an international student who applied for “hundreds of positions without receiving even one interview” in his first year (FG). Research positions, in particular, are in high demand for students interested in pursuing graduate studies or research work.

Students involved in the program also feel that there is a general lack of awareness in the undergraduate population about the program. Participants note that the main way of learning about it is through emails and newsletters, which students often disregard. In contrast, those who attained positions report hearing about them through informal means. For example, Janice was recommended by her friend for her library job (LM). Another international student, Arjun, comments that it may have helped that his campus employer attended the same college in his home country (FG). Kayla was recommended by a friend to a campus lab job (FG). Jenny, a domestic student, learned about the campus program from her older brother, who urged her to apply in her first year (FG). She applied for 30 jobs and was interviewed for one.

In contrast, Wendy, a first-generation university student (a student whose parents did not attend university), comments that none of her friends knew about the campus program, adding that she was fortunate to find out about a summer position through a professor who “reached out to me” (FG). Another first-generation student, Charlotte, describes the application process as “pushing myself out of my comfort zone” (FG). Salima, a first-generation student who is also an international student, attended campus events to meet researchers who hire students in order to improve her chances of being hired. Therefore, it is clear that students who lack connections must be more proactive and may need more encouragement to pursue such positions.

However, students who are required to fund most of their studies are also likely to lack the necessary time to secure campus employment, especially if prior volunteer work is expected. Such students are required “to be actively and critically evaluating demands on their time” (Billett, 2015, p. 147). For example, a common pathway for science students, as Drew shares, involves volunteering first at a lab in the hopes of moving into a paid position. The tactic of volunteering as an entrée to paid work is mentioned by another student, Prakash, who learned about a volunteer opportunity that led to paid employment through his academic advisor (LM). In contrast, a student with a disability felt that applying for campus work would require more time than she could afford, and her past experiences discouraged her. Students who self-fund their education are typically unable to limit their hours to 10 per week and must work multiple jobs. For example, in the summer after her second year, Liz worked at a grocery store for 20 h per week, a campus job at a student café for 10 h per week, and enrolled in an internship for course credit that required 14 h of work per week (FI). As per program regulations, students can only hold one campus position at a time.

In sum, while a number of students count themselves lucky because they have secured campus employment, it is clear that social connections and confidence to seek out opportunities are key factors too. Also, there are not enough positions to meet student demand. Therefore, equitable access to campus employment program jobs is an important issue.

### The relevance of term-time work for studies and career plans

Given that several students see a disconnect between their campus jobs and studies, it is important to examine the reasons for this perception and look at whether students regard it as problematic. We look at the role of academic program and occupational norms in student expectations about work. We also consider why students appear to value campus jobs even when they aren’t seen as congruent with their studies and career aspirations. Our analysis confirms differences in students’ positions and dispositions (Hodkinson et al., 2007) which influence their approaches to gaining career-relevant work. First-generation students, students with unclear aspirations, and some students who lack prior work experience (often international students) are less likely to seek career-relevant campus jobs early in their degrees. For the latter two groups, motivations to work are often driven by a desire to secure any paying job whatsoever, to begin to build their resumes and to learn about work culture. In contrast, domestic students from professional families and students with more concrete career and mobility plans appear to worry more about career-relevance early on in their degrees. Through time, the majority of students express a desire for more “useful” (Isabelle), “applicable,” (Dana), and “specific” (Penny) term-time work. Examining changes in students’ interests and work experiences throughout their program addresses the limits of cross-sectional work-study role research (Butler, 2007).

For example, it is clear that most students see term-time work as only one of many possible ways of gaining experience in their fields over their degree; other vehicles include volunteer work, extra-curricular activities, off-campus work, and cooperative education. This leads to another key insight: students who do not perceive work-study congruence still value campus employment highly for other reasons, most notably, their workplace relationships, flexibility, and contrast with studies. Supportive relationships with co-workers and supervisors help students feel integrated into the larger campus community. In some cases, campus jobs provide a relief from the stress of studies and a convenient entrée into the work world. Almost universally, students in campus jobs laud their worker-centred flexibility (Chung et al., 2013). These themes are elaborated below.

### Work-study congruence

Role theory assumes that work-study congruence is good and leads to WSF (Owen et al., 2017). However, our interviews suggest that work-study congruence is more important for some students than others, WSC and WSF are multifaceted, and differences in student positions and dispositions impact work-study relationships (Hodkinson et al., 2007). For example, if students are unclear about their studies and career plans, tight connections between term-time work and studies are less likely to be a priority. Further, if students are engaged in a wide range of activities besides paid work and studies, connections between them may not be as critical (cf. Billett, 2015). The following portraits of students indicate the complexity of work-study relationships.

Wendy comes from an immigrant family without university-educated parents and has worked since high school. Although she is uncertain about career plans, after securing one summer campus job related to her studies, she continued working year round at a retail store off-campus for the rest of her degree. Her apparent lack of concern about work-study linkages might be attributed to uncertainty about where her studies will lead. Although she works in a “very white-female dominated company,” she places a high value on comfort and friends in work (FG). In contrast, she describes university as “confusing … being surrounded by people all the time who have such a specific goal in mind and all know exactly what they want to do after their undergrad makes me feel like maybe I should find out soon” (FG). Wendy’s comfort with little work-study congruence might be explained by the pressure she admittedly placed on herself to fund her degree herself and graduate without debt (FI). Thus, she worked part-time from September to April and typically worked each summer at a couple of retail and coffee shop jobs for 40 to 50 h per week.

While she was also uncertain about her career plans, Rose was confident that her wide range of campus experiences were preparing her for future work. These campus experiences involve a highly competitive campus job, volunteer work with a campus residence, and various extra-curricular activities. It is perhaps unsurprising that she too appears comfortable with a lack of direct work-studies connection because she is in a general program (Arts), has little prior work experience as an international student, and wants to explore: “I feel like at university that’s where I can really put those feelers out, and make sure that I find something that I am good at, and find something I love and that I can then follow in the future” (FG). Strong parental support and the fact that she gained permanent resident status during her degree (which lowered her tuition dramatically) no doubt contribute to her confidence.

Our interviews with Rose and others indicate further that many working students adopt a “portfolio approach” to career preparation: they participate in volunteer work, cooperative education, undergraduate research, and extra-curricular activities, in addition to studies and campus jobs. This recalls Feher’s (2009) discussion of contemporary approaches to human capital, which see individuals as “managers of a portfolio of conducts pertaining to all the aspects of their lives” (p. 30). For example, Charlotte comments, “I’m happy that I have this job so I don’t really have to worry too much about trying to do a club thing” to get experience in her field (FG). Similarly, Ranbir is content to work at a recreation job on campus because he expects to gain career relevant experience through cooperative education placements as part of his business program. This portfolio approach is not captured by work-study research that centres the impact of paid work on academic outcomes.

In contrast, another international student, Ting, is in an Arts program where unpaid internships are the norm, “so we kind of understand that we have to start off without the intent of earning money” (FG). Seeking “hands on” experience in the local film industry means working on-call for long hours, since securing better, unionized work requires permanent resident status. In comparison, Ting’s campus job seems uncomplicated and reliable. While Judith is also in a campus job with clear connections to her program of study, it seems to spark a different kind of work-study conflict (WSC). For example, Judith describes her campus job,

[W]e would incorporate whatever teaching concepts we are doing into activities or projects. So, we teach them [youth] chemistry by going through a chemistry experiment and explaining things as we go, instead of teaching them, “okay you need to know this, you need to be able to calculate this.” We skip the boring stuff [laughter] and have fun. (LM)

She then compares it to her studies,

[W]e have design projects that we have to do in class anyways, like we have a six-credit design project course thing this term that I'm not taking because I failed the pre-req last term, but the thing about that is that you're under the pressure of getting graded based on how well you do. And it's like, you have to make it do certain things, and it's like you don't have as much freedom and creativity. (FG)

Judith’s work appears to conflict with her studies in that it makes her more critical of them; this is a different take on the idea that work is taking away resources from studies (i.e., WSC) (Butler, 2007). For students like Judith, work is regarded as a “nice way to de-stress” from school, which sparks important questions about what work-study facilitation means from a student perspective. For example, for international students who lack work experience prior to university, WSF may involve learning about work in Canada while moderating the pressure of academic acculturation. Janice, a Southeast Asian international student shares that she desires a job that would help her ease into a work routine (LM). Like Judith, she appreciates that her campus position provides space to forget about her competitive academic program.

For three other international students (two male and one female), the stakes of work decisions appear higher, and they engage with campus paid and unpaid work, studies, and other activities with a level of energy and drive that stand out in our study. All three are racialized students from lower-middle income countries in applied degree programs, and all reference the hard work required to get to Canada and the pressure of financial insecurity and family expectations. Equally hard working, albeit in a less focused way, are four female domestic students with significant retail and service work experience from high school. They compare their “good” university jobs favorably with these “bad” high school jobs. For example, Fiona recognizes that her campus job as peer support for other students sometimes takes a toll on her wellbeing, but notes that her campus work has also increased her expectations and standards for future work: “the people I surround myself with, and just the expectations I have of employers and employees… I know what it’s like to work in a [good] environment.”

While these four young women seem to internalize the pressure to find career-related work, in a follow-up interview one of them expresses ambivalence about the idea that students should only focus on building skills for a career. During her undergraduate program, Liz moved from an off-campus job with little flexibility or opportunities for advancement to a campus job that is closely related to her studies. She deliberately made this transition “from working just to make money and make ends meet and working to develop my skills that will be useful for me in my degree” (FI). At the same time, her comments about remote, computer-based work during the pandemic suggest the complexity of choices for students,

[A]s much as I was excited to get away from working … at a grocery store, having what I study and what I do for work being the same thing became very overwhelming for me. You know, like, not having a job where I could just do something a bit more mindless and enjoyable, became really stressful. So, I think there is value in having separate sections in your life and like, throughout your degree working in something that's not necessarily specifically related to what you’re studying is not always a bad thing. (FI)

The discussion above suggests that cross-sectional research on work-study roles misses changes in students over time as well as the multiplicity of factors that affect their decisions and outcomes, including financial security and student mobility. Thus, WSF and WSC can be complicated. Literature on learning cultures directs our attention to the ways in which students are embodied beings who are socially positioned and in a process of continual becoming (Hodkinson et al., 2007, 2008). Family background and resources, prior experiences with work, academic program norms, and expectations of students and their families can be seen to influence students’ approaches to term-time work in different ways over time. Also important, of course, are the affordances of different worksites. The next section addresses these affordances and the question of why students whose campus jobs are not seen as relevant to their studies and career aspirations continue to value them highly.

### The value of supportive work relationships and flexibility

As noted, our 30 participants tend to define work that is related to studies more expansively than “jobs that develop career skills.” Most suggest that their relationships at work are key to how they feel about it, and in turn, how it impacts their studies. Student workers are shaped by and shape practices in their worksites (Hodkinson et al., 2008). Focusing on the 15 students who perceived their campus jobs to be disconnected or only partially connected to their studies/aspirations, we find that most value their campus jobs for the quality of their workplace relationships and flexible working conditions. In most of these jobs, students work alongside peers, and this fact, coupled with employers who recognize their student status, is perceived to facilitate their academic work (cf. Butler, 2007). In particular, most of the 15 students see relationships with peer co-workers as key to their wellbeing, and supervisors as providing valued flexibility. For the few students who worked independently, supervisory relationships are more important.

Several students refer to the importance of relationships with peers. For example, Ranbir recalls a time when he “had a tiff with a few workers” which negatively affected his motivation to go to work at his recreation job. However, he was later able to find a “like minded” group, and at the time of our audio diaries, reports enjoying his friends at work. In fact, he plans to keep working at this job because of these friendships. Another international student, Isabelle, describes her work as a friendly “community” based on teamwork and support, which is a nice change from the individualized competition in her academic courses (FG). A third international student, Dana, also refers to her campus job as a good way to meet people on a large and busy campus (FI).

Similarly, Bradley, a domestic student from an immigrant family, sees his work in recreation primarily as a place to develop a peer support network while making money,

When I go to work, it sort of feels like it’s a social time, sort of in a good way because it makes time pass… As we do our jobs the way we have to, there’s a lot of time to talk to each other and to just learn about each other’s lives. And there’s a lot of people that maybe are taking the same courses or have taken the courses that you had before. So, not only is it a job, but it's also really good to meet new friends. (FG)

Connor, a domestic first-generation student, plans to stay in his ‘disconnected’ campus job because of its “friendly” environment (FG). Like Ting, he does not expect to find paid career-relevant work during his general degree, and believes that “you have to kind of pay your dues” before finding professional work.

As one of few students who was working on her own, Kay speaks about the importance of her supervisor,

He’s been so lovely to me as I try to navigate the university and all the mental health things. He’s really been above and beyond as some kind of mentoring or advisor role. Just because the university is so big that there is no automatic mentor advisor person here for you, so it was just really nice having that contact. And if there is one thing, I have to say I got out of that job, it was probably my relationship with my boss. (LM)

In addition to relationships, the majority of the 30 campus employment participants value the flexibility afforded by their employers. They appreciate the ability to build their work schedule around their academic schedule, work schedules that are consistent, being able to work from home, and (although less common) being able to vary their work times according to the rhythms of the semester. In short, most participants feel grateful that they are able to prioritize their academics. Two (overlapping) groups are particularly appreciative of such flexibility: those with prior experience working off-campus in low-wage service jobs, and students working in campus jobs that are less connected to their studies.

Connor, who represents both groups, shares,

[My campus job] is probably the best job I have ever worked. It’s a lot more chill than my other jobs were and it gives me the time I need to focus on my classes, because I don’t ever have to worry about them conflicting at all. If I have a midterm coming up, I can just let them know and they won’t schedule me. … I am used to 40 hour [a week] jobs [in summer], so working 12 hours is nothing.

Thus, his prior experience in fast-paced service jobs off-campus makes him appreciate worker-centred flexibility.

Regina, another domestic student working in campus recreation finds flexibility at work when she is able to do some academic readings or practice questions (FG). During the pandemic, some campus employers allowed remote work or a combination of in-person and work-from-home arrangements. Instead of directly controlling students’ time, they trusted them to regulate when they work and how they distribute their time. Thus, for some participants, campus positions provide the benefits and job satisfaction found in upper-level jobs without the intensity of these positions (Kossek & Lautsch, 2018). During the pandemic, of course, the challenges of remote work and studies was also evident as students’ bedrooms became their workspace (Taylor, 2022a). Still, in contrast to off-campus jobs, campus positions are required to recognize and accommodate students’ academic commitments by offering flexibility in work (cf. Hill et al., 2008).

Students are cognizant that the flexibility of campus employment positions is uncommon in most student jobs. As Heather says, “I know not a lot of people have that kind of flexibility where they can say they’re going to start work any time and just start doing it” (AD). Like Connor, Rajesh compares his work on- and off-campus,

[In my campus job], they say, “Yeah, academics is priority.” They don’t even allow you to work more than nine hours, you know. They have those rules. … [Whereas at my current off-campus job] if I have a shift on Monday, I cannot skip it until I find a co-worker to take it on. If I do skip it, it looks bad, or I have to call and be like, “I am sick.” (FG)

While the actual work he’s doing in the two jobs is similar, flexibility makes the difference. Ranbir too continues to work at his campus job despite lack of work-study congruence precisely because it allows him to prioritize his studies (FG). Finally, until Blake has the necessary knowledge to find work related to his studies, he is satisfied with his library job (one of his three jobs) because it allows him to slow down his busy work-study life, “you just kind of do a little bit of quiet, simple tasks, and everything’s quiet around you, and you just do that for a couple of hours” (FG).

The preceding examples suggest the importance of worker-centred flexibility (Chung et al., 2013) for students. Further, their positions and dispositions impact the kind of flexibility and work relations they seek over time. For example, Salima is financially insecure and felt pressure to gain career-relevant experience early in her degree to facilitate her application to a professional program in the future. However, she felt the need to adjust her plans when her ‘connected job’ became exhausting,

I still want to work next year, but I’m hoping to look for a job that is not very structured in terms of hours, because that would let me have a little bit more flexibility. For example, you know when midterms are rolling in and you have so many assignments due, it would be easier to have work that isn’t so structured so that I can take time out and prioritize. I would also try and look for work where there is a balance between physical and mental, you know, energy. You know right now I feel like the work I am doing– it’s mentally exhausting, and so I think I would try and find a neutral balance there if I can. (AD)

Thus, in addition to career-relevant skills, students are clearly learning through term-time work how to pace themselves during the school year and over their degrees (cf. Taylor, 2022b).

In sum, although campus employment jobs vary in terms of the extent of work-study congruence for different students, most provide a high degree of flexibility for students. Since a number of participants in the campus program work multiple jobs (many for financial reasons), further research should explore the cap on hours and the implications of the requirement that students work the same number of hours each week. While there is clearly no one-size-fits-all, most students desire work flexibility that recognizes the rhythms of the academic semester.

## Concluding comments

The reason why I continued to work [at campus job] was just because the environment is really nice and I'm friends with basically everyone at work … We all hang out together. We grab food together, hang out a lot during the summer. So it's like, we're all just like, “hey, are you gonna be here next term and if you are, I'm gonna stay too!” (Judith, FG)

Overall, our interviews with students suggest that campus employment jobs are perceived to have the edge over jobs off-campus beyond the professional ‘skills building’ reasons promoted by programs. Supportive relationships with co-workers and employers are critical components of such programs, especially for students who see their work as disconnected or only partially connected to their studies. While some students seek work-study congruence, others are looking for jobs that reduce the stress of academics, help them acculturate to a large campus, or learn about the Canadian work context. Overall, for our “time jealous” students (Billett, 2015), flexibility and control are crucial; the ability to decide how to organize work while prioritizing academics is valued highly in campus employment. Further, regulations like the cap on weekly hours and consistency in hours per week increases the appeal of these jobs.

Still, it is clear that one size does not fit all in terms of what ‘good’ jobs look like for students and there is variation in the trade-offs they are required to make. We can better understand differences in the student experience by attending to the interaction between what students bring to work (including prior work experiences, positions, and dispositions) and the affordances of different workplaces, as Hodkinson et al. (2007) suggest. For example, we find that students with prior experience in low-wage service jobs are most likely to value the flexibility and regulation of campus employment, but may lack the confidence or connections needed to apply. Our research highlights that longitudinal mixed methodology research can address some of the limitations of cross-sectional survey research and work-study role literature. For example, data from our 30 participants provides a sense of how students think about WSC and WSF. It also indicates that they adopt a portfolio approach to gaining experience which often includes unpaid work and campus clubs. We further contribute to the literature on student roles by highlighting that work-study relationships are bi-directional (see Wyland et al., 2016), complex, and fluid. Student dispositions change over time as they clarify where they want to go and how to get there. Further research on campus employment programs should continue to explore how working students’ family background and resources, prior experiences with work, academic program norms, aspirations, and the affordances of different worksites influence their experiences and outcomes.

Study findings have implications for university staff including employers and staff in campus employment programs. Our analysis suggests that most students perceive workplace relationships to be as important as learning skills, given the demands of their programs and challenges in navigating a large campus. To help more students make connections with their studies, programs could make a point of providing paid work time for students to engage in reflection exercises that help them make explicit what they are learning (Billett, 2015). This is in keeping with other research which argues for more structured learning support (West & Stirling, 2021). More institutional research could be beneficial in exploring challenges for financially insecure students. It could also examine student demographics by position category to address inequities in access. Finally, universities should consider different ways for campus employers to engage in dialogue with student workers around their aspirations and constraints, and should support students in advocating for their rights on- and off-campus. As an educational institution and employer, the university is well-situated to take a leadership role in public education and critical advocacy around matters of working conditions and workplace relations.

## Data Availability

The datasets generated and analysed during the current study are not publicly available. This was not a requirement of the granting agency at the time of the award. Further, since the dataset is part of research that is longitudinal and involves current students, sharing it could compromise the anonymity of participants.
